# Human peripheral blood mononuclear cells enriched in endothelial progenitor cells via quality and quantity controlled culture accelerate vascularization and wound healing in a porcine wound model

**DOI:** 10.1177/0963689718780307

**Published:** 2018-07-05

**Authors:** Makiko Kado, Rica Tanaka, Kayo Arita, Kayoko Okada, Rie Ito-Hirano, Satoshi Fujimura, Hiroshi Mizuno

**Affiliations:** 1 Department of Plastic and Reconstructive Surgery, Juntendo University School of Medicine, Tokyo, Japan

**Keywords:** Chronic wounds, endothelial progenitor cells, peripheral blood mononuclear cells, porcine wound model, vasculogenesis, wound healing

## Abstract

The transplantation of endothelial progenitor cells (EPCs) is used to promote wound angiogenesis. In patients with chronic wounds and accompanying morbidities, EPCs are often compromised in number and function. To overcome these limitations, we previously developed a quality and quantity controlled (QQ) culture system to enrich peripheral blood mononuclear cells (PBMNCs) in EPCs. To evaluate the wound healing efficacy of mononuclear cells (MNCs) harvested after QQ culture (QQMNCs), preclinical studies were performed on large animals. MNCs harvested from the blood of healthy human subjects were cultured in the presence of angiogenic cytokines and growth factors in a serum-free medium for 7 days. A total of 5 × 10^6^ QQMNCs per full-thickness skin defect or control saline was injected into wounds induced in cyclosporine-immunosuppressed pigs. EPC colony-forming assays revealed a significantly higher number of definitive (partially differentiated) EPC colony-forming units in QQMNCs. Flow cytometry evaluation of QQMNC surface markers showed enrichment of CD34^+^ and CD133^+^ stem cell populations, significant reduction in CCR2^+^ cell percentages, and a greater than 10-fold increase in the percentage of anti-inflammatory M2-type macrophages (CD206^+^ cells) compared with PBMNCs. Wounds treated with QQMNCs had a significantly higher closure rate. Wounds were harvested, frozen, and sectioned at day 21 postoperatively. Hematoxylin and eosin staining revealed that the epithelization of QQMNC-treated wounds was more advanced than in controls. Treated wounds developed granulation tissue with more mature collagen and larger capillary networks. CD31 and human mitochondrial co-staining confirmed the presence of differentiated human cells within newly formed vessels. Real-time polymerase chain reaction (PCR) showed upregulation of interleukin 6 (IL-6), IL-10, and IL-4 in the wound bed, suggesting paracrine activity of the transplanted QQMNCs. Our data demonstrate for the first time that QQ culture of MNCs obtained from a small amount of peripheral blood yields vasculogenic and therapeutic cells effective in wound healing.

## Introduction

Neovascularization is one of the most important factors in the granulation and remodeling phases of wound healing^1^ as it provides nutrients and oxygen to all participating metabolically active cells^2,3^. Promoting vasculogenesis and angiogenesis is therefore critical for successful wound healing.

De novo vasculogenesis occurs through two different processes: vasculogenesis, the de novo formation of blood vessels from endothelial progenitor cells (EPCs), and angiogenesis, the expansion and sprouting of new blood vessels from existing blood vessels. Previously, vasculogenesis was thought to occur only during fetal development, but ever since the discovery of EPCs by Asahara et al. in 1997, it has been reported that vasculogenesis occurs in adults as well^4^. Since then, multiple attempts have been made to utilize EPCs to improve wound healing^5^.

Patients with chronic conditions, such as diabetes^6,7^ or severe ischemia^8^, have been reported to yield fewer EPCs, the vasculogenic functions of which were found to be weaker than those from healthy individuals.

We previously described a new method for generating EPCs with enhanced vasculogenic and angiogenic potential. Mononuclear cells (MNCs) harvested from peripheral blood are cultured in vitro with our quality and quantity controlled culture (QQ culture) system in the presence of stem cell factor (SCF), thrombopoietin, vascular endothelial growth factor (VEGF), interleukin (IL)-6, and Flt-3 ligand^9,10^. MNCs harvested after QQ culture (QQMNCs) showed enrichment of CD34^+^, CD133^+^, and CD206^+^ cell populations with increased numbers of colony-forming units (CFU), while the numbers of several hematopoietic cell types, including B lymphocytes, proinflammatory monocytes/macrophages, and natural killer cells, were decreased^11^.

This study aims to preclinically validate the effectiveness of EPCs generated by QQ culture for wound healing in a porcine wound model.

## Materials and Methods

### Serum-Free QQ Culture

Experiments using human samples were performed with institutional approval and following guidelines from the Ethical Review Board of Juntendo University School of Medicine (No. 2017016). All volunteers provided informed consent to participate in this study. Blood was drawn (50 mL) using a heparinized syringe via forearm venipuncture of 10 healthy volunteers, aged 30–40 years (mean: 35.7 years). Peripheral blood MNCs (PBMNCs) from each donor were isolated via density-gradient centrifugation using a Lymphocyte Separation Solution (d = 1.077; Nakalai Tesque, Kyoto, Japan). A final concentration of 2 × 10^6^ PBMNCs was seeded in each well of a six-well plate (BD Falcon, Bedford, MA, USA) and cultured at 37°C and 5% CO_2_ in a serum-free QQ culture for 7 days without changing media, as described previously^10^. QQ culture consisted of serum-free Stemline II medium (Sigma-Aldrich, St. Louis, MO, USA) supplemented with an optimized growth factor/cytokine mixture of 20 ng/mL thrombopoietin, 20 ng/mL IL-6, 100 ng/mL SCF, 100 ng/mL Flt-3 ligand, and 50 ng/mL VEGF (all from Peprotech, Rocky Hill, NJ, USA). PBMNCs cultured for 7 days in QQ culture were termed QQMNCs. Prior to QQ culture, MNCs were labeled with PKH26 Red Fluorescent Cell Linker Kit (Sigma-Aldrich) according to the manufacturer’s instructions.

### EPC Colony-Forming Assay

Fresh PBMNCs derived from healthy volunteers and corresponding QQMNCs were seeded at a concentration of 2 × 10^5^ cells/dish in 35-mm Primaria dishes (BD Falcon) and cultured for 16–18 days using the EPC-CFA system (Methocult SF^BIT^; STEMCELL Technologies Inc., Vancouver, BC, Canada). The semi-solid culture medium was supplemented with a growth factor/cytokine mixture of 66.7 ng/mL SCF, 33.3 ng/mL VEGF, 33.3 ng/mL basic fibroblast growth factor (FGF), 33.3 ng/mL EGF, 33.3 ng/mL insulin-like growth factor 1 (IGF-1), 13.3 ng/mL IL-3 (Peprotech, Rocky Hill, NJ, USA), 1.33 IU/mL heparin (Shimizu Pharmaceutical Co., Shizuoka, Japan), and 30% fetal bovine serum (SAFC Biosciences, St. Louis, MO, USA). After 16–18 days in culture, the colonies in each dish were enumerated under an inverted microscope (Nikon Corporation, Tokyo, Japan) at 40× magnification, as described previously^10^. Two types of EPC colony-forming units (EPC-CFU) were observed and determined: primitive EPC-CFU (pEPC-CFU) and definitive EPC-CFU (dEPC-CFU)^5,12,13^.

### Flow Cytometry

A total of 5 × 10^5^ cells were resuspended in 200 μL of phosphate buffered saline (PBS) containing 2 mmol EDTA and 0.2% bovine serum albumin (EDTA/BSA/PBS) and were incubated with 10 μL of human FcR blocking reagent (Miltenyi Biotec, Bergisch Gladbach, Germany) at 4°C for 30 min. The cell suspension was equally divided into reaction tubes and incubated at 4°C for 20 min with 2 μL of the following antibodies: CD206-PE/Cy7, CD3-Alexa700, CD4-FITC, CD8-Pacific Blue, CD14-APC/Cy7, CD192(CCR2)-PerCP/Cy5.5, CD184(CXCR4)-PE/Cy7 (BioLegend, San Diego, CA, USA), CD133-APC (Miltenyi Biotec), CD34-PE, and CD31-FITC (BD Pharmingen, Franklin Lakes, NJ, USA). After incubation, cells were washed twice with 1 mL of EDTA/BSA/PBS, and flow cytometry was performed using FACSAria™ III (BD Biosciences, Franklin Lakes, NJ, USA). The data were analyzed using Flowjo software (Tomy Digital Biology Co. Ltd, Tokyo, Japan).

### Porcine Wound Model

**Figure 1.**
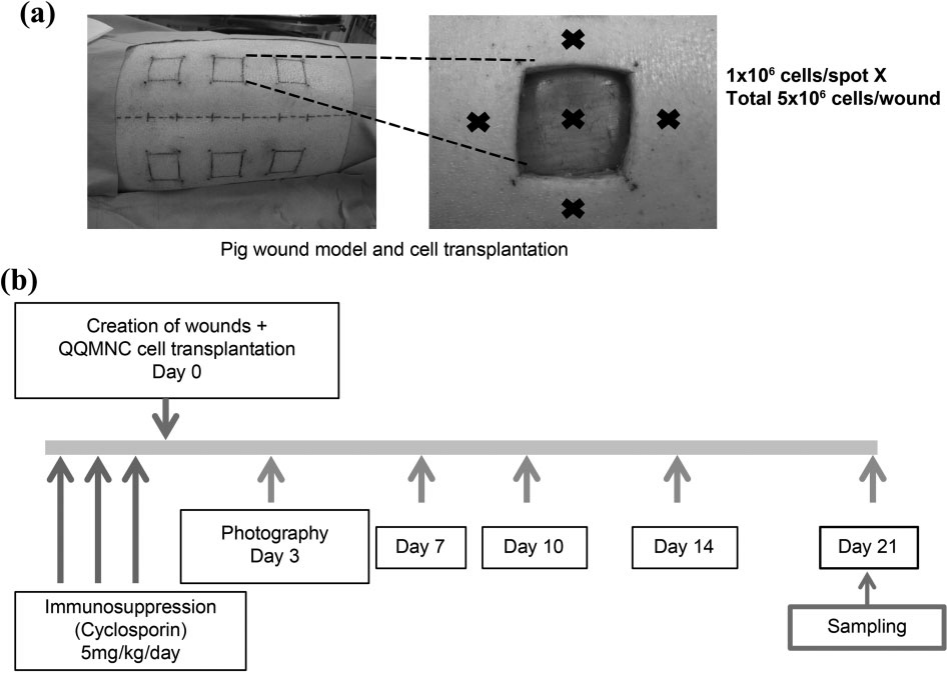
Porcine wound model and time schedule for wound observation. (a) Six full-thickness skin defects (measured 2.5 × 2.5 cm^2^) were induced on the back of each pig. Wounds were separated from each other and the backbone by 3 cm. QQMNCs were injected intramuscularly into five spots within each wound at 1 × 10^6^ cells/spot. The remaining three wounds were injected with saline as control. (b) After wound induction and injection of QQMNCs, wounds were photographed at the indicated time points. On day 21, the wounds were excised, frozen, and sectioned. QQMNC: Mononuclear cells harvested after quality and quantity controlled culture.

In total, seven male LDW breed pigs (Landrace × Large White), weighing 20.4–27.4 kg (mean ± SD: 22.7 ± 2.193 kg), were purchased from Shiraishi Animals Co. Ltd. (Saitama, Japan). All experimental procedures were carried out in accordance with the guidelines specified by the committee of Ethical Animal Care and Use at Juntendo University School of Medicine. For immunosuppression, 5 mg/kg/day of cyclosporine (Neoral solution 10%; Novartis Pharmaceuticals, Tokyo, Japan) was administrated 3 days before cell transplantation. The pigs were first sedated through intramuscular injection of a mixture of xylazine (2 mg/kg of body weight; Bayer Yakuhin, Ltd., Tokyo, Japan) and ketamine (20 mg/kg of body weight; Kyosomirai Pharma Co. Ltd., Tokyo, Japan), followed by inhalation of 4% isoflurane (Pfizer Japan Inc., Tokyo, Japan) for general anesthesia, after which the wound area on the back of the animals was depilated. Two days later, the pigs were anesthetized again using the same protocol. The skin surface was sterilized with 10% povidone-iodine solution (Yoshida Pharmaceutical Co. Ltd, Tokyo, Japan) followed by sodium hyposulfite in alcohol, and then six full-thickness epifascial skin defects sized 2.5 × 2.5 cm^2^ were induced 3 cm apart (Figure 1(a)). Crystal violet (Alfresa Pharma Co., Osaka, Japan) was applied intradermally at the margins in order to permanently mark the wound edges (Figure 1(a)).

### QQMNC Cell Transplantation Therapy

Each pig received a total of six wounds. Three wounds on each animal were treated with saline, and the other three wounds with either QQMNCs or PKH26-labeled QQMNCs. For cell treatment, 1 × 10^6^ QQMNCs or PKH26-labeled QQMNCs in 250 µL saline were injected intramuscularly at five different spots in and around the wound (a total of 5 × 10^6^ cells; Figure 1(a)), using a 1-mL syringe with a 27-gauge needle. Each wound received cells from one individual donor. For the control group, 250 µL of saline was injected intramuscularly. Thereafter, the wound area was covered with a sterile gauze dressing, polyurethane film, Autohesion bandage (Coban 3 M, Tokyo, Japan), and an elastic bandage (Kawamoto Co. Ltd, Osaka, Japan). For infection control, 0.1 g/kg/day of amoxicillin-clavulanate potassium (GlaxoSmithKline K.K., Tokyo, Japan) was administered for 3 days postoperatively.

### Postoperative Wound Observation and Harvesting

Wounds were photographed on days 0, 3, 7, 10, 14, 17, and 21 after QQMNC transplantation (Figure 1(b)). Wound photographs were acquired with an 8-megapixel digital camera (Canon USA, Melville, NY, USA) from a sufficient distance to include the whole wounded area (9 × 7 cm^2^) and with the lens oriented parallel to the wound. The wound area was measured photogrammetrically (Photoshop CS3; Adobe Systems, San Jose, CA, USA) and the percentage of wound closure was calculated as [1 − (current wound area/original wound area)] × 100%.

Wounds were harvested from anesthetized pigs at day 21 postoperatively. Wounds were excised at a thickness of 3 cm from the surface layer to include the muscle layer. Excision was made 1 cm beyond the margin of the original wound edge, which was demarcated with crystal violet. Each sample was vertically divided into four blocks. Sample blocks were frozen in optimal cutting temperature compound (Tissue-Tek; Sakura Finetek Japan Co. Ltd, Tokyo, Japan) for cryo-sectioning either directly or after gradual dehydration with 5% to 25% sucrose solution in 4% paraformaldehyde. Sections (8 µm thick) were cut from the central region of the wound, which allowed the observation of capillary vessel formation.

### Hematoxylin-Eosin (H-E) Staining to Measure Epithelialization Gap

The 8 µm thick sections were fixed with 10% formalin and stained with H-E according to a standard protocol. After staining, the sections were photographed at 40× magnification using a light microscope (BZ-9000; KEYENCE, Osaka, Japan) and combined to create a picture of the whole wound using image analysis software (BZ-analyzer; KEYENCE). The epithelial gaps were measured photogrammetrically.

### Van Gieson Staining for Evaluating Wound Maturity

Stained sections were observed using a light microscope at 40× magnification. Pixelated areas were measured using image analysis software (BZ-analyzer), and the percentage of mature collagen was calculated by dividing strongly red areas (mature collagen) by pale to strongly red areas (total collagen area).

### CD31 Staining for Vascular Endothelium

Sections (8 µm thick) were fixed with 4% paraformaldehyde, and antigen retrieval was performed using proteinase K (Dako Japan, Tokyo, Japan) according to the manufacturer’s protocol. After 1 h of blocking with PBS containing 10% goat serum, the sections were incubated overnight with mouse anti-pig CD31-FITC antibody MCA1746F (Bio-Rad, Hercules, CA, USA) diluted 1:50 with PBS containing 2% goat serum and 0.05% Triton X-100. Sections were observed at magnifications ranging from 50× to 630× using a fluorescent microscope (DM400B; Leica, Wetzlar, Germany) and photographed with a high-sensitivity digital color fluorescence camera (DFC310 FX; Leica, Wetzlar, Germany) at 20× magnification. CD31-positive vessels were counted using image analysis software (BZ-analyzer). Ten fields under the superficial layer and 10 fields in the middle layer of the granulation tissue were enumerated and used to determine the average number of vessels per 200× magnification “field of view” (FOV).

### Anti-Human Mitochondrial Antibody Staining of the Transplanted QQMNCs

Sections were fixed with 4% paraformaldehyde, and antigen retrieval was performed using proteinase K (Dako, CA, USA) according to the manufacturer’s instructions. After 1 h of blocking with PBS containing 10% goat serum, the sections were incubated overnight with anti-human mitochondrial antibody (HMA), MAB 1273, clone 113 -1 (Merck Millipore, Darmstadt, Germany) diluted 1:200. For HMA detection, mouse anti-goat Alexa Fluor® 594 antibody (Thermo Fisher Scientific, Waltham, MA, USA) diluted 1:1000 in PBS containing 2% goat serum and 0.05% Triton X-100 was used. Finally, the cells were co-stained with mouse anti-pig CD31-FITC antibody, as described previously, and DAPI (Vector Laboratories, Burlingame, CA, USA). Sections were observed at magnifications ranging from 50× to 630×, using a fluorescent microscope and photographed with a high-sensitivity digital color fluorescence camera. The number of CD31-positive vessels (green) and double-positive CD31/HMA/PKH26 vessels (orange) was determined using image analysis software (BZ-analyzer), and the percentage of vessels containing human cells was determined.

### Real-Time Polymerase Chain Reaction (PCR)

Total RNA was extracted from control wounds and those injected with QQMNCs. The material for RNA isolation was obtained from the muscular layer (1 cm from the inferior edge of each wound where QQMNCs were injected) of harvested frozen wounds using a 6-mm biopsy puncher (Maruho, Osaka, Japan). Biopsy material was placed in TRIzol solution and homogenized with a tissue homogenizer (Bio Masher II; Nippi, Tokyo, Japan). RNA was extracted by adding chloroform, 100% isopropanol, and 75% ethanol. Reverse transcription was performed using a High-Capacity RNA-to-cDNA kit (Applied Biosystems, Foster City, CA, USA). cDNA was added to a reaction master mix (TaqMan FAST Universal PCR Mastermix, Thermo Fisher Scientific) and amplified using the StepOnePlus Real-Time PCR system (Thermo Fisher Scientific). PCR mixtures were preincubated at 95°C for 20 s, followed by 45 cycles of 95°C for 1 s and 60°C for 20 s. The data were analyzed using the change in threshold cycle (Ct) method (relative Ct method). ΔCt was calculated as (target gene Ct) − (βactin Ct), and the relative quantity of target gene mRNA was determined using 2^−ΔCt^. TaqMan probes (Applied Biosystems) for the target porcine genes are listed in Table 1.

**Table 1.** List of TaqMan probes for Real-Time PCR.

Gene	Catalog No.
βactin	Ss03376081_u1
TGFβ	Ss03376081_u1
FGF	Ss03375684_s1
IL-4	Ss03394126_m1
IL-6	Ss03384604_u1
IL-10	Ss03382372_u1

### Statistical Analysis

GraphPad Prism (GraphPad Software, La Jolla, CA, USA) was used for all statistical analyses. Data were presented as the mean ± standard deviation (SD). Statistical significance was assessed using Student’s *t*-test and Wilcoxon signed-rank test. Statistical significance was set at p < 0.05.

## Results

### Effects of QQ Culture on MNCs Isolated from Peripheral Blood of Healthy Individuals

**Figure 2.**
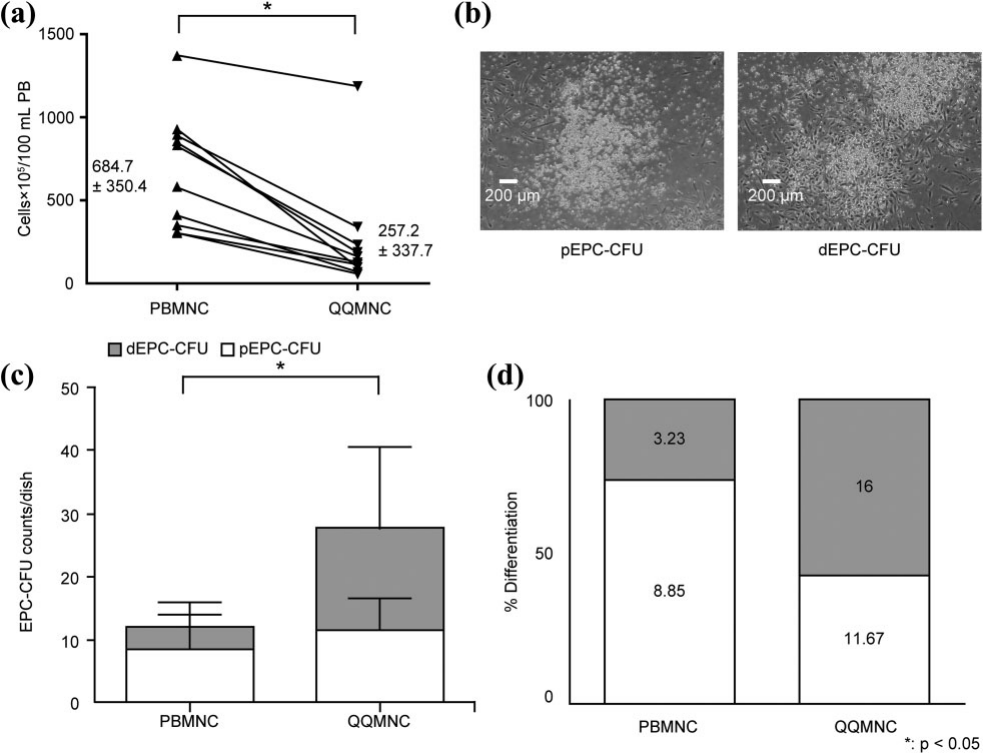
Characterization of untreated MNCs (PBMNCs) and QQ culture MNCs (QQMNCs). (a) PBMNC and QQMNC counts per 100 mL of peripheral blood. PBMNC *n* = 10, QQMNC *n* = 10. (b) Example of pEPC-CFU (left) and dEPC-CFU (right). 40× magnification FOV. (c) Quantification of total EPC-CFUs including pEPC-CFUs and dEPC-CFUs generated from PBMNCs or QQMNCs plated at 2 × 10^5^ cells/dish. Each bar represents mean ± SD, PBMNC *n* = 7, QQMNC *n* = 4. *p < 0.05. PBMNC total CFU versus QQMNC total CFU. (d) Percentage differentiation into pEPC-CFUs and dEPC-CFUs based on their respective average counts (from Figure 2(c) and included inside the bars). dEPC-CFU: definitive endothelial progenitor cells colony-forming units; PBMNC: peripheral blood mononuclear cells; pEPC-CFU: primitive endothelial progenitor cells colony-forming units; QQMNC: mononuclear cells harvested after quality and quantity controlled culture.

For quantity control, the number of freshly isolated MNCs (PBMNCs) was compared with the number of QQMNCs from the same donor after QQ culture. The number decreased from 644.7 ± 100.8 × 10^5^ of PBMNCs to 337.7 ± 106.8×10^5^ of QQMNCs per 100 mL of blood (p < 0.05; *n* = 10; Figure 2(a)).

To evaluate the vasculogenic potential of QQMNCs, an EPC-CFU assay was performed. EPC-CFUs can be classified as pEPC-CFUs formed by small round cells and dEPC-CFUs formed by larger spindle-like cells (Figure 2(b)). In pEPC-CFU colonies, cells have higher proliferation rates, whereas cells in dEPC-CFU colonies are more differentiated and more adherent^12,13^. QQMNCs produced a larger number of dEPC-CFUs, 16 ± 12.94 vs. 3.23 ± 1.85, and significantly increased total EPC-CFU counts, 27.67 ± 9.65 vs. 12.08 ± 8.54 (p < 0.05; Figure 2(c)) than PBMNCs. Consequently, the ratio between the more mature dEPC-CFUs and pEPC-CFUs shifted towards more mature dEPC-CFUs in QQMNC dishes, where the proportion of dEPC-CFU became 57.8% compared with 26.7% in control PBMNC dishes (Figure 2(d)).

To further characterize QQMNCs, surface expression of stem cell markers and markers related to angiogenesis was analyzed using flow cytometry. The percentage of cells expressing the stem cell marker CD34 (0.21 ± 0.24 vs. 1.35 ± 0.62, p < 0.05) was more than six times higher, and the percentage of cells expressing the stem cell marker CD133 (0.09 ± 0.08 vs. 0.25 ± 0.10, p < 0.05) was 2.5 times higher in QQMNCs than in PBMNCs. There was no significant difference between the two groups in the number of cells expressing the endothelial cell marker CD31 (59.52 ± 9.40 vs. 55.45 ± 8.37). The percentage of CCR2^+^ cells (pro-inflammatory monocyte/macrophage population that expresses CC chemokine receptor 2) drastically decreased in the QQMNC group (22.80 ± 7.09 vs. 1.74 ± 1.05, p < 0.05), while the percentage of anti-inflammatory M2-type macrophages (CD206^+^ cells) increased more than 10-fold compared with PBMNC (1.04 ± 2.06 vs. 13.11 ± 7.35, p < 0.05). In addition, we observed a significant increase in CD4^+^ helper T cells (p < 0.05; Table 2).

**Table 2.** Flow cytometry analysis of stem cell and angiogenesis-related markers in PBMNCs and QQMNCs. % in PBMNC, the percentage of positive cells in the PBMNC population; % in QQMNC, the percentage of positive cells in the QQMNC population. The expression of each marker in two populations was compared using a Student’s *t*-test; p values are shown, and data are presented as mean ± SD of *n* = 6 experiments.

Surface marker	% in PBMNC	% in QQMNC	p Value
CD34^+^	0.21 ± 0.24	1.35 ± 0.62	0.0313
CD133^+^	0.09 ± 0.08	0.25 ± 0.10	0.0313
CD206^+^	1.04 ± 2.06	13.11 ± 7.35	0.0313
CD31^+^	59.52 ± 9.4	55.45 ± 8.37	0.1563
CD3^+^	50.99 ± 9.27	62.14 ± 12.43	0.0625
CD4^+^	32.93 ± 3.78	47.69 ± 8.94	0.0313
CD8^+^	15.19 ± 4.57	17.06 ± 6.01	0.4375
CD14^+^	19.28 ± 11.07	14.29 ± 9.20	0.0625
CCR2^+^	21.04 ± 5.96	0.89 ± 0.70	0.0313
CD3^+^/CXCR4^+^/CD31^+^	27.14 ± 7.43	37.69 ± 11.63	0.0313

### QQMNC Therapy Accelerates Wound Closure

**Figure 3.**
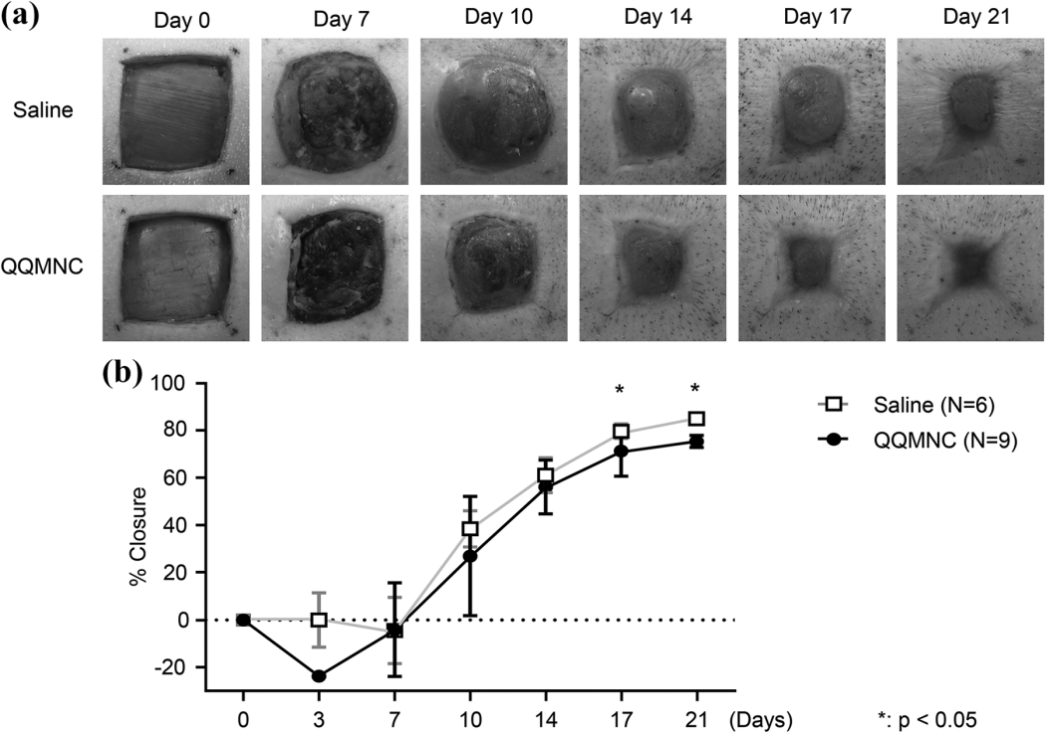
Comparison of wound closure rates in QQMNC-treated and saline-treated wounds. (a) Wounds were photographed at the indicated time points. Wounds tended to epithelialize faster in the QQMNC-transplanted group than in the saline-treated control group. (b) Wounds were photographed on days 0, 3, 7, 10, 14, 17, and 21. Wound area was measured photogrammetrically, and the percentage of wound closure was calculated as [1 − (current wound area/original wound area)] × 100%. Each bar represents mean ± SD, saline *n* = 6, QQMNC *n* = 9 wounds. QQMNC: mononuclear cells harvested after quality and quantity controlled culture.

To investigate the effect of QQMNCs on the rate of wound healing, wounds were photographed at various time points post-injection, and the wound area was measured photogrammetrically. Significantly larger wound areas achieved better closure in wounds treated with QQMNCs than with saline (control group). On day 10, the percentage of wound closure was 44.33 ± 14.01% for QQMNC-treated wounds vs. 26.10 ± 29.43% for control wounds. The percentage of wound closure in QQMNC-treated wounds as compared with control wounds on days 14, 17, and 21 was 64.20 ± 10.92% vs. 55.96 ± 1 3.41% (p > 0.05), 80.26 ± 4.41% vs. 70.04 ± 10.74% (p < 0.05), and 85.04 ± 0.96% vs. 75.39 ± 2.58% (p < 0.05), respectively (Figure 3).

### QQMNC Transplantation Accelerates Wound Epithelialization

**Figure 4.**
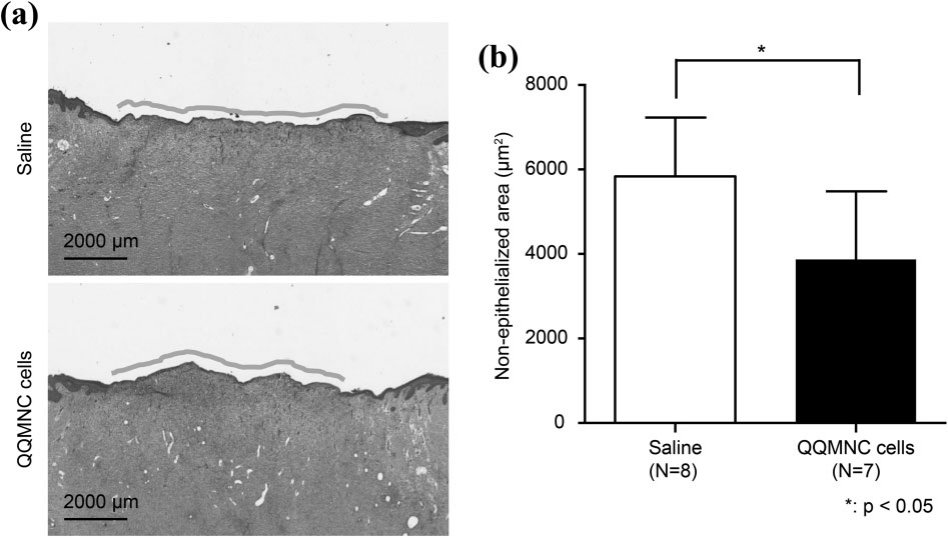
Wound epithelialization. Hematoxylin and eosin (H-E) staining was performed to evaluate epithelialization. (a) Microscopic images of the H-E stained wounds. (b) Quantitation of the non-epithelialized areas of the wounds. Each bar represents mean ± SD, saline *n* = 8, QQMNC *n* = 7, 40× magnification FOV per wound. QQMNC: mononuclear cells harvested after quality and quantity controlled culture.

H-E staining was performed to investigate whether QQMNC treatment improves epithelialization (Figure 4(a)). The epithelial gap in wounds treated with QQMNCs was significantly smaller than in saline-treated wounds, at 3870.9 ± 1613.9 µm vs. 5835.1 ± 1391.74 µm, respectively (p < 0.05; Figure 4(b). No other epithelial differences were observed between QQMNC-treated and saline-treated wounds.

### QQMNC Therapy Accelerates Granulation Tissue Maturation

**Figure 5.**
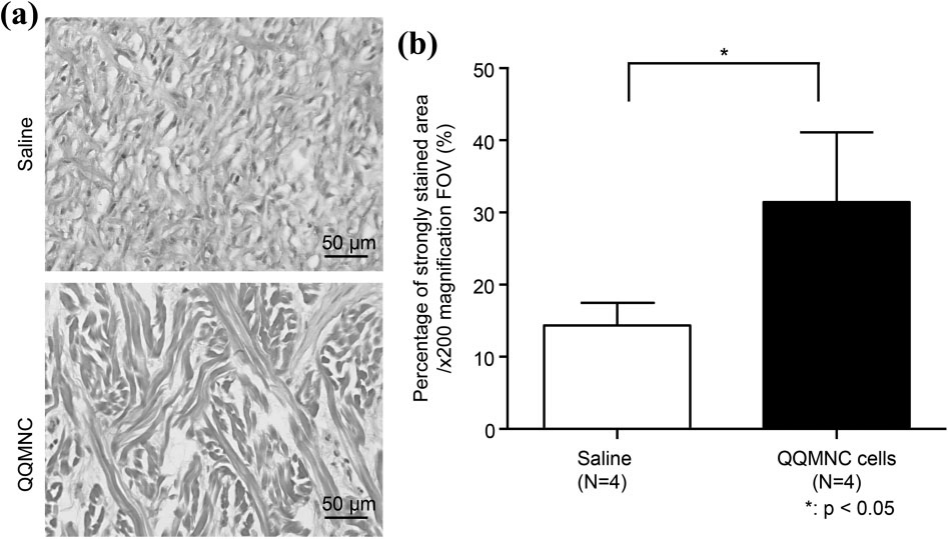
Visualization and quantitation of mature collagen by van Gieson staining. (a) Representative microphotographs of wound sections after van Gieson staining. High-density, thick, and strongly stained collagen fibrils are seen in the QQMNC-treated group on day 21 after treatment. (b) Percentage of the area stained strongly with van Gieson staining. Percentage of mature collagen was calculated by measuring the red pixelated area. Each bar represents mean ± SD, saline *n* = 4, QQMNC *n* = 4, 200× magnification FOV. QQMNC: mononuclear cells harvested after quality and quantity controlled culture.

Granulation tissue was examined by van Gieson staining for mature collagen. Thick, high-density collagen fibers stained bright red were observed in QQMNC-treated wounds compared with the control saline-treated wounds, where the staining was faint (Figure 5(a)). The percentage of mature collagen was determined by measuring the red pixelated areas. The area of mature collagen was significantly larger in QQMNC-treated wounds than in control, at 31.45 ± 4.82 vs. 14.36 ± 1.57, respectively (p < 0.05; Figure 5(b)).

### QQMNC Therapy Promotes Vascularization

**Figure 6.**
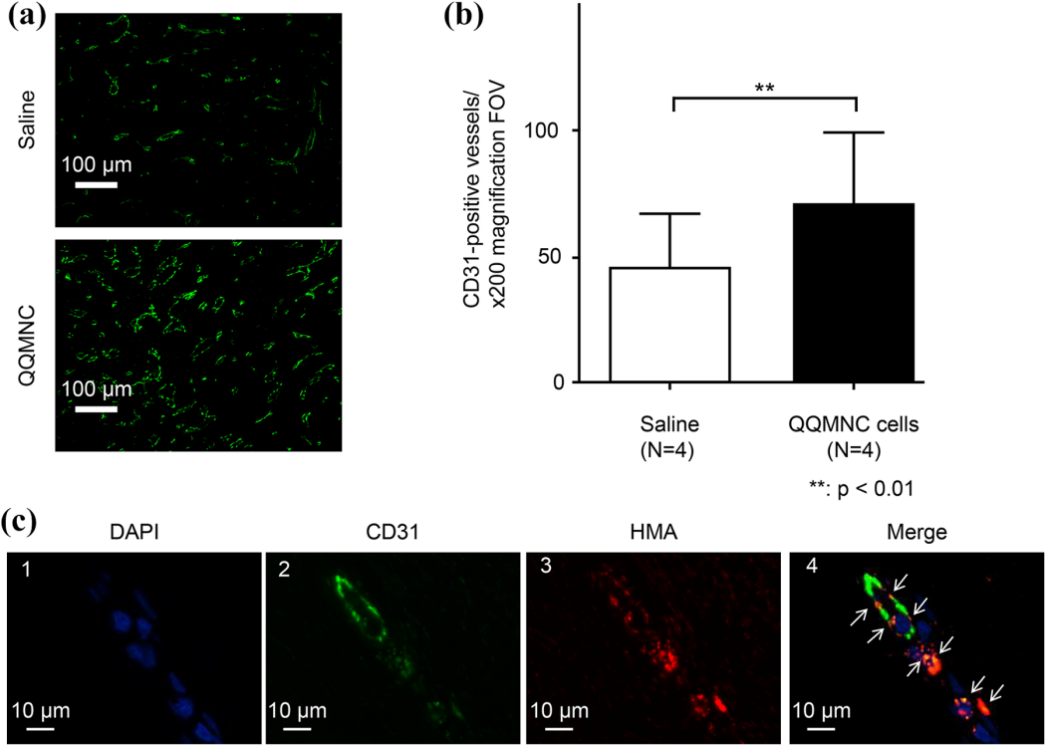
Visualization and quantitation of capillaries in QQMNC-treated and control wounds by staining with the anti-CD31 antibody. (a) Representative microphotograph of granulation tissue in wound sections of control (top) and QQMNC-treated (bottom) wounds stained with the anti-CD31 antibody; 200× magnification. (b) Quantification of the number of capillaries. Significantly more CD31-positive vessels were present in QQMNC-treated wounds compared with saline-injected wounds. Each bar represents mean ± SD, saline *n* = 4, QQMNC *n* = 4, 200× magnification FOV. (c) Assessment of inclusion of human cells into newly formed capillaries in the muscular layer 1 cm from the edge of wounds injected with PKH26-QQMNC. 1: DAPI staining. 2: CD31 staining. Endothelial cells are stained in green. 3: Human mitochondria staining. The PKH26-QQMNC red signal is enhanced by HMA staining. 4: Merged microphotographs 1, 2, and 3. White arrows point to overlapping CD31 and HMA staining (orange) suggesting that QQMNC differentiated into endothelial cells.

To visualize the newly formed capillary vessels in granulation tissue, the sections were stained with an antibody against endothelial marker CD31. A more extensive capillary network with wider vessels was observed in the microphotographs of wounds treated with QQMNCs when compared with those of control saline-treated wounds (Figure 6(a)). Quantification of capillary vessels demonstrated a significantly greater number of capillaries in the QQMNC-treated group compared with the control group, at 71.2 ± 27.49 vs. 45.3 ± 21.69, respectively, per 200× magnification FOV (p < 0.01; *n* = 12; Figure 6(b)).

### Inclusion of Human EPCs in Newly Formed Capillary Vessels

To investigate whether injected QQMNCs participated in the formation of capillaries, sections of wounds injected with labeled QQMNCs (PKH26-QQMNCs) were stained with anti-CD31 antibody and HMA to enhance PKH26 staining of human cells. Cell nuclei were visualized using DAPI (Figure 6(c)1). Histological observations of stained sections showed that injected human cells were present in the muscle layer (1 cm from the inferior edge) of the wound. Microphotographs of muscle layer identified CD31-stained cells (green; Figure 6(c)2) and PKH26/HMA-stained cells (red; Figure 6(c)3). When these microphotographs were merged, PKH26/HMA-stained cells completely overlapped with CD31-positive cells, demonstrating orange color (Figure 6(c)4), suggesting that QQMNCs differentiated into the endothelial cells. The percentage of the area of red-stained human cells in the newly formed capillary vessels stained with CD31 antibody was 25.91 ± 6.02% (*n* = 12).

### QQMNCs Activate Gene Expression of Porcine Growth Factors, Cytokines, and Surface Markers Related to Wound Healing and Vasculogenesis in the Wound Bed

**Figure 7.**
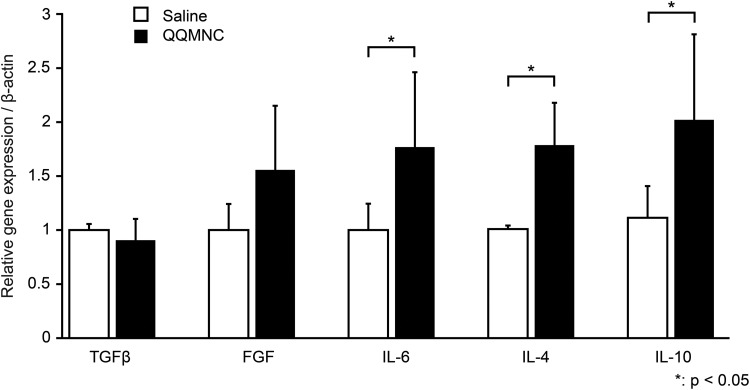
Gene expression evaluation of growth factors and cytokines related to wound healing and vasculogenesis in the wound bed. As demonstrated through real-time PCR analysis, expression of IL-6, IL-4, and IL-10 was significantly increased in QQMNC-treated wound tissues compared with saline-treated controls. Each bar represents mean ± SD; TGFβ: QQMNC/saline *n* = 10/6, FGF: QQMNC /saline *n* = 5/5, IL-6: QQMNC /saline *n* = 6/5, IL-4: QQMNC /saline *n* = 6/4, IL-10: QQMNC /saline *n* = 6/5.

To evaluate paracrine activities of injected QQMNCs, RNA was isolated from the wound tissue, and real-time PCR of relevant porcine genes was performed. The results are shown in Figure 7. A significant increase in gene expression of inflammation-related cytokines, IL-6 (1.7-fold), IL-4 (1.79-fold), and IL-10 (2.02-fold), was observed in QQMNC-treated wounds compared with saline-treated wounds. The gene expression of growth factor FGF also increased (1.56-fold); however, this increase was not statistically significant. No significant increase in the gene expression of transforming growth factor beta (TGFβ) was observed (Figure 7).

## Discussion

Adequate blood flow is crucial for wound healing. Since the administration of endothelial progenitor cells was recently suggested for treating chronic wounds^14,15^, two major methods of EPC treatment have been developed. The first method is the non-selective transplantation of EPCs that form a fraction of the hematopoietic stem cells harvested directly from bone marrow or through apheresis from peripheral blood (after recruitment of hematopoietic stem cells with granulocyte colony-stimulating factor (G-CSF))^16^. The other is a selective method that requires isolation of hematopoietic stem cells based on surface markers CD34 and/or CD133 from bone marrow or peripheral blood samples^17–20^. However, current treatments with EPCs face several problems. Patients with chronic wounds often have associated comorbidities, such as cardiac and lung disease, ischemia, endocrine, gastrointestinal, and hematologic disorders, and musculoskeletal problems, as well as alcohol/drug abuse, smoking, the use of immunosuppressive drugs, cancer chemotherapy, and steroids. EPC yields in the peripheral blood of such patients are low, and the angiogenic and vasculogenic potential is diminished^4,5,21^.

To overcome these limitations, we have developed a less invasive and less laborious method to obtain larger quantities of EPCs with increased vasculogenic potential.

Although MNC numbers decreased by approximately 30% after QQ culture, QQMNCs were enriched with CD34^+^ and CD133^+^ EPC populations (stem cell markers). Additionally, the treatment of MNC in QQ culture resulted in a larger CD206^+^ anti-inflammatory M2 macrophage population, while the number of pro-inflammatory macrophages expressing the chemokine receptor CCR2 drastically decreased. One can speculate that these changes in macrophage polarization contribute to the effect of QQMNCs on wound healing.

QQ culture produced significantly higher numbers of partially differentiated definitive colonies in CFU assays. dEPC-CFUs are considered more suitable for clinical applications due to their more potent vasculogenic and angiogenic activities^13^.

In the present preclinical study, we investigated the effects of QQMNCs in a xenograft porcine wound model, as QQ cultures require growth factors not available for pig cells.

Since the current study was performed in preparation for a clinical trial, the investigation of QQMNC properties in a large animal was preferred. Pigs were chosen as the model for this study because porcine organs, muscle, and bone are similar to those of humans in physiology and size, and the swine itself is an appropriate mass to model that of an adult human. Porcine skin is relatively hairless compared with other large experimental animals, and its structure and turnover cycles are similar to those in humans. Not only does wound healing occur in physiologically similar phases in pigs and humans (inflammation, proliferation, re-epithelialization, and remodeling), but pigs^22^ also form scars in a somewhat similar manner to humans^23,24^. Our model is based on previous studies that have used porcine excisional wound models^25–27^.

We initially theorized that immunosuppression of the pigs might inhibit immune cells, such as macrophages and T-cells, that actively participate in wound repair^21^. Hence, we produced both control saline-treated wounds and QQMNC-treated wounds on each animal and characterized wound healing. Surprisingly, we were able to demonstrate QQMNC efficacy in immunosuppressed animals.

In contrast to previous reports suggesting that immunosuppression could inhibit vasculogenesis^24,28^, our model achieved wound closure with newly formed blood vessels within 3 weeks. QQMNCs accelerated the formation of granulation tissue, enhancing the production and maturation of collagen. The size of collagen fibers in the QQMNC-transplanted group was also greater than that in the control group. There was also increased blood vessel formation in the QQMNC-transplanted group compared with the control.

Our data indicate that the promotion of vasculogenesis triggered by QQMNCs plays a key role in the enhancement of wound healing. Visualization and quantitation of human cells incorporated into newly formed blood vessels by CD31 and HMA co-staining confirmed the existence of viable, proliferating, and differentiating EPCs within the QQMNC population. To exclude the possibility of false-positive HMA staining of wound sections, HMA staining was performed in sections extracted from the control wounds treated with saline. The results were negative (data are not shown). In addition, the fact that HMA staining completely overlapped with CD31-positive cells further supports our conclusion that HMA-positive human cells differentiated in vivo into endothelial cells.

The ultimate goal for the clinical use of QQMNCs is their administration in patients with slow-healing or non-healing chronic wounds. However, the production of chronic wounds in animals, particularly in large animals, is a challenging task. The full-thickness epifascial wounds produced in our study were observed for 3 weeks, which was long enough for the wound to be considered slow-healing. In comparison, recent studies on the surgical treatment of chronic wounds in similar porcine models show that most of the healing and treatment effects occurred by 3 weeks, though the animals were observed for 8 weeks^29^. Our results confirm our previous findings that QQMNCs formed tubular structures in vitro and blood vessels in vivo in an ischemic hind limb mouse model^11^. Further, a full-thickness model was chosen, and not, for example, a hind limb ischemic model, because our goal was to investigate not only the effect of QQMNCs on vasculogenesis, but also their paracrine effect on wound closure.

Previously, several studies reported positive paracrine effects of mesenchymal stem cells (MSCs) and CD34^+^ cells on wound healing via secretion of cytokines such as FGF, VEGF, and various matrix metalloproteinases (MMPs) to promote vascular endothelial regeneration^30^. Similarly, EPCs were found to promote wound healing by paracrine upregulation of fibroblast and keratinocyte migration^31^. Masuda et al. reported upregulation of anti-inflammatory cytokine IL-10, and other genes related to angiogenesis and tissue regeneration, such as VEGF, IGF, MMP-2, and MMP-9, in QQMNCs^11^. Our study demonstrated that in addition to their direct involvement in vasculogenesis, QQMNCs caused indirect paracrine upregulation of several porcine mediators known to participate in wound healing, such as IL-6, IL-4, IL-10, and FGF. Since the wounds were excised at 3 weeks after QQMNC injection, we were unable to evaluate the expression of human mediators directly produced by QQMNCs, which peak within a few hours to several days after wound infliction^32^. However, our results suggest that the observed acceleration of angiogenesis, epithelization, collagen synthesis, and wound closure in QQMNC-treated wounds can be explained by a paracrine effect of transplanted QQMNCs. Molecular mechanisms underlying the effect of QQMNCs on fibroblast proliferation and collagen synthesis are currently under investigation.

Our preliminary studies showed that the quality and quantity controlled culture system for treating non-healing wounds in human subjects requires only 200 mL of peripheral blood, which can be obtained via a standard venipuncture method in an outpatient clinic. At least 2 × 10^7^ MNCs can be isolated from 200 mL of peripheral blood, enough to produce 20 injections of 10^6^ cells required for wound healing in humans, in accordance with our previous report^33^. The QQ culture does not contain animal products or serum, does not require changing of media or re-plating, and can be easily adjusted for clinical use. Overall, the QQ culture method is non-invasive, cost-effective, and more efficient in obtaining viable EPCs capable of proliferation, differentiation, and participation in vasculogenesis and angiogenesis in vivo. In addition, QQMNC therapy can potentiate other cellular mechanisms that participate in wound healing.

## Conclusion

QQ culture of PBMNCs produces vasculogenic and therapeutic cells from a small amount of peripheral blood. Our study is the first to demonstrate the efficacy of QQMNC treatment in wound healing as a preclinical study. This treatment method is a potentially cost-effective and non-invasive alternative to the current EPC treatment of chronic wounds. At present, we are working on a transportation system for QQMNCs, which, when established, could make treatment with QQMNCs clinically available worldwide. Based on the promising results described here, we are currently conducting a phase-I clinical study of QQMNC transplantation for chronic wounds, to analyze its safety and efficacy as a treatment method.
